# Does It Matter to Establish a Strategic Partnership for COVID-19 Prevention and Control? The Perspective of Multiple Distances in Emerging Economies

**DOI:** 10.3389/fpubh.2022.894816

**Published:** 2022-07-14

**Authors:** Can Zhao, Meng-Yang Wei, Yan Chen, Ruo-Yan Shen

**Affiliations:** School of Economics and Management, Beijing University of Posts and Telecommunications, Beijing, China

**Keywords:** pandemic prevention and control, strategic partners, multiple distances, emerging economies, COVID-19

## Abstract

The rapid spread of the COVID-19 pandemic in early 2020 has impacted the politics, economy and society of countries around the world. The public health diplomacy system through which developed countries in Europe and America used to provide vertical one-way assistance to developing countries faces huge challenges. How emerging economies can cooperate to fight the pandemic on the basis of mutual trust and mutual benefit has become an urgent issue. In this paper, we examine the impact of political mutual trust on the effectiveness of pandemic prevention and control from the perspective of establishing strategic partnerships between emerging economies. Furthermore, taking into account the huge differences between emerging economies, this paper explores institutional distance, cultural distance, and geographical distance—the adjustment effect of the control effect. Studies have shown that the improvement of political mutual trust is conducive to the formation of a community of shared futures between countries and has a positive effect on curbing the spread of the pandemic. The increase of the three-dimensional distance of institutions, culture, and geography will weaken the effect of establishing strategic partners for pandemic prevention and control. This paper explores a new model of horizontal international cooperation among emerging economies, and provides a reference for emerging economies to deal with common globalization issues in the future.

## Introduction

Since the beginning of 2020, the rapid spread of COVID-19 has brought a great impact on the world's politics, economics, and society. Different from the major public security incidents for human beings, in the face of this COVID-19 pandemic, there are huge differences in the prevention and control strategies of the world's major economies. U.S. states have introduced different pandemic prevention policies, such as issuing stay-at-home orders, closing schools and public places, canceling public events, and others (1). The British government adopted a “risk aversion” policy and launched a plan to contain, delay, research and mitigate in the fight against the pandemic, trying to prevent and control it through herd immunity. Countries such as China and South Korea have adopted strong government interventions such as urban lockdowns, mass virus testing, and case isolation to control the spread of the virus (2).

In response to the outbreak, countries have increased public health spending to different degrees. According to preliminary WHO estimates, per capita health spending in 22 countries (mostly developed countries) increased in real terms by 4.9% in 2020, well above the average annual growth rate of 2% between 2017 and 2019, and Canada's per capita COVID-19 spending was as high as $602 (3). According to statistics from Johns Hopkins University in the United States, of the 10 countries most affected by the pandemic, seven are developed countries^1^ As a result, the public health diplomacy system in which developed countries in Europe and America provided vertical and one-way assistance to developing countries in the past will face enormous challenges. In this context, it is extremely important to “hold together for warmth” through international cooperation among emerging economies (4) with low economic levels and a lack of medical facilities.

International cooperation is critical in addressing global health and public health security concerns, and is key to the response to the COVID-19 pandemic (5). The essence of pandemic prevention and control is comprehensive behavior involving multiple tasks (6) related to vaccine production, transportation of pandemic prevention materials, and cross-border flow of personnel and service elements. The urgency of controlling the spread of the virus has put forward higher requirements for the facilitation of customs clearance between countries, the liberalization of investment and trade, cultural inclusion, and scientific data sharing. The improvement of bilateral and multilateral political mutual trust will have a positive role in promoting bilateral trade, and will be more conducive to the formation of a new community of national economic interests and a community of destiny. For example, during this pandemic, emerging economies in the South Pacific region established a regional cooperation mechanism to deal with the pandemic—the Pacific Humanitarian Pathway on COVID-19 (PHP-C)—which strengthened the political level to respond to the pandemic. Regional cooperation has accelerated the clearance of medical supplies and medical personnel, delaying or mitigating outbreaks in the Pacific (7).

Previous studies have explored how to prevent and control the spread of the epidemic from multiple perspectives, including public health management (8), clinical features and pathological mechanism research (9, 10), vaccines and diagnostic methods (11, 12), transmission models (13), the auxiliary application of information technology (14) and international cooperation (15). With respect to research literatures on international cooperation, many literatures emphasized that scientific collaboration in the field of health plays an important role in reducing the infection rate and mortality of infectious diseases (16–20). In addition, some literatures analyzed the prevention and control of infectious diseases from the perspective of the construction of global health governance mechanisms (21, 22). However, only a few articles focused on the impact of relations among states on international cooperation to solve the global public health crisis (23). At present, there is little empirical evidence on whether the strategic partnership of emerging economies based on political mutual trust has a positive effect on pandemic prevention and control.

The unique urgency and uncertainty of major public emergencies have not only impacted the global public health system, but also provided opportunities for countries to cooperate. This paper takes into account the large number and wide distribution of emerging economies and the huge institutional and cultural differences between countries, which may lead to differences in their stressors, responses, and priorities (24). These differences greatly increase the difficulty of coordination and may adversely affect the effectiveness of pandemic prevention and control. Therefore, this paper incorporates the geographical distance, institutional distance and cultural distance between countries into the research framework, explores a new model of horizontal international cooperation among emerging economies, and provides new ideas for emerging economies to deal with global issues such as public health events, climate, and resources in the future.

## Theoretical Underpinning and Hypotheses Development

Strategic cooperative partnership between countries means mutual trust, more channels of interaction and institutions established to solve problems at the governmental and non-governmental levels. In the face of global crises and challenges, countries can transcend bilateral issues, seek common ground while reserving differences, and seek international cooperation for common solutions (25). Therefore, establishing a strategic partnership can effectively improve the situation of the large-scale spread of the pandemic by strengthening international cooperation in pandemic prevention and control, and ultimately reduce the number of new coronary pneumonia infections and deaths. In terms of improving the effectiveness of pandemic prevention and control, the role of establishing strategic partners is mainly reflected in the following two aspects. First, governments should strengthen mutual trust and enhance bilateral political relations in order to promote active public health diplomacy between countries and reduce political obstacles to cooperation in pandemic prevention. They should avoid government decision making that may terminate certain freedoms and powers in the name of national security because of the public health securitization program (26). Such political intervention in the interest of national self-interest will reduce resources for the global response to the public health crisis. Second, they should provide a platform for researchers to conduct joint research, reduce scientific and vaccine nationalism, and work together to find effective treatments for COVID-19. The existence of scientific nationalism will lead to many political challenges for international cooperation (19)—for example, accusing some countries of stealing COVID-19 research data, deliberately spreading misinformation, conducting international competition on vaccines, etc. (27–29). Vaccine nationalism will make it difficult for emerging economies with weaker economic and technological capabilities to have equitable access to COVID-19 vaccines (30), thus preventing the spread of the disease from being effectively contained. Based on the above analysis, this paper proposes the following hypotheses:

H1: Establishing cooperative partnership plays a positive role in pandemic prevention and control.

The dimensions of differences between countries are diverse, and the institutions, cultures, and geographical distances between countries may have an impact on the international cooperation carried out under the partnership, which in turn makes the establishment of strategic partnerships have different influences on the effect of pandemic prevention and control.

Institutions include formal institutions (e.g., law and regulations) and informal institutions (e.g., self-imposed codes of conduct) (31), and institutional distance is the degree of institutional difference between home and host countries (32). In the work of pandemic prevention and control, political and institutional arrangements in various sectors of society such as finance, communications, media, and diplomacy are very necessary, and it is not enough to rely solely on biomedical prevention and control (33). The greater the institutional distance, the greater the differences in rules and norms between countries, which may be detrimental to the prevention and control of the cross-border spread of the COVID-19 pandemic.

On the one hand, the existence of institutional distance makes it difficult for countries to reach consensus on divergent issues, which may increase political tensions and make international cooperation in prevention and control more difficult (34). Mutual support and cooperation in the political field will promote national cooperation in various fields such as economy, science and technology, medical and health care, and it will be easier for the two sides to actively cooperate during the pandemic and provide each other with humanitarian assistance. For example, political realities determine the scope and approach of scientific collaboration, and tense political relations may lead to stagnation in the sharing of COVID-19 data and research findings (20). On the other hand, the existence of institutional distance may lead to differences in the measures taken by countries in pandemic prevention and control and their implementation, which in turn has an adverse impact on the effectiveness of prevention and control. For example, in the early stage of the outbreak of COVID-19, China implemented the strictest closed control and social isolation, established a strict prevention and control system with the participation of the whole people, and effectively blocked the chain of virus transmission through non-drug means (35). However, there are also some countries, subject to their own political systems and legal mechanisms, where it is difficult to take measures to enforce the isolation of high-risk groups and the forcible prohibition of high-risk behaviors.

H2: Institutional distance negatively regulates the positive effect of establishing strategic partners on pandemic prevention and control.

There are cultural differences between different countries (36), and cultural distance represents the degree of those differences (37). The smaller the cultural distance, the higher the cultural similarity and cultural recognition between two countries. Compared with countries with larger cultural distances, two countries with the same or similar values will have closer exchanges and cooperation in pandemic prevention and control. First, countries are more inclined to provide humanitarian aid to other countries that are closer to their own culture. For example, Turkic-speaking countries are more likely to receive Turkish medical assistance (23). Turkey's selective strategy of providing medical aid to the world shows that cultural distance is one of the important factors influencing a country's foreign aid policy. Second, individual behavior and decision-making are largely influenced by national culture. Successful measures and experiences in one country's response to the spread of the pandemic are more applicable to other countries that are consistent with their own cultural values. When responses to public health problems are not adapted to the local context, they can backfire (33). For example, a cultural atmosphere that pays more attention to individual rights and freedoms has caused some people in the United States to ignore the mask ban, and the resulting protests and demonstrations have led to crowds gathering, increasing the risk of COVID-19 transmission. Third, cultural distance increases the transaction costs associated with cross-cultural communication, leading to a reduction in international scientific research cooperation in pandemic prevention and control (38). However, the collaboration of researchers from different countries on COVID-19 virus research is more conducive to obtaining diverse knowledge and information and stimulating innovative ideas (39).

H3: Cultural distance negatively regulates the positive effect of establishing strategic partnerships for pandemic prevention and control.

The difficulty of national cooperation is increased by increasing geographical distance, which is challenging to interrupt the rapid spread of COVID-19 around the world. On the one hand, geographic distance is a negative factor affecting state relations and is also seen as a proxy for transportation and communication costs (40), which not only adds obstacles to material assistance, but also increases transaction costs for medical care supplies. On the other hand, geographical distance can affect people's cognitive attitudes toward the pandemic, and only when people are geographically concerned about the virus will they behave more consciously to comply with preventive measures (41). This means that although other countries face serious COVID-19 threats, due to their geographical distance, it is difficult for their own people to recognize the seriousness of the problem, resulting in a lack of response measures and a strong desire to engage in international cooperation. At the same time, the regional organization formed by the geographical relationship can effectively promote the participation of various economies in the region in regional health diplomacy and governance (42). Because the population and commodity circulation are the most dense and frequent between countries that are geographically close to each other, there are relatively complete economic, political, cultural, and other comprehensive cooperation mechanisms between regions. Based on the existing cooperation foundation, it is easier for countries to cooperate in the field of public health. In the face of a global public health crisis, cooperation between neighboring countries can effectively curb the spread of the pandemic to surrounding areas. Previous researchers have found that research cooperation on COVID-19 shows highly regionalized characteristics, with some geographically close countries tending to be more active in international cooperation (43, 44).

H4: Geographical distance negatively regulates the positive effect of establishing strategic partners on pandemic prevention and control.

## Data Sources and Specification of Variables

### Data Sources

There is not yet a consistent definition of the concept of emerging economies, and a common practice is to classify countries other than developed as emerging economies (45, 46). First of all, this article confirms the list of 197 sovereign states in the world, including 193 UN member states, two UN observer states (Vatican City, Palestine), and two non-joined UN states (Niue, Cook Islands). Secondly, one of the evaluation criteria for developed countries is to join the Organization for Economic Cooperation and Development (OECD) (47). However, due to the different evaluation indicators of different international institutions, it is controversial whether some countries in the OECD are developed countries. Therefore, this paper chooses 32 developed countries recognized by both the United Nations and the OECD. Finally, after excluding 32 developed countries, this paper obtained 165 emerging economies including China as the research object. Considering that China is the largest emerging economy in the world, and has established extensive cooperative partnerships with other emerging economies—including 39 comprehensive strategic partner countries, 21 strategic partner countries, and six comprehensive partner countries^2^—its rich multi-level strategic cooperative partnership is very suitable for the research in this paper.

We collected China's strategic partnership with 164 emerging economies in 2019, and cross-sectional data on the pandemic, economy, population, and medical care of these countries in 2020. We mainly considered two aspects. One is the availability of data. In 2020, the COVID-19 virus spread widely around the world and was identified by the World Health Organization as a public health emergency of international concern. In addition, some indicator statistics in the World Health Organization and World Bank databases are only updated to 2020. Second, the impact of close strategic partnership on the effectiveness of COVID-19 prevention and control has a lag. The data for each indicator used in this article is taken from the Johns Hopkins University Coronavirus Resource Center, the website of the Chinese Ministry of Foreign Affairs, the public data of the Hofstede team, the CEPII database, the World Bank, the World Health Organization, and the United Nations Comtrade Database.

### Variables Selection

#### Dependent Variable

The number of confirmed cases is one of the important indicators to reflect the effectiveness of a country's pandemic prevention and control (15). The fewer the number of confirmed cases, the better the effectiveness of that prevention and control. Therefore, this paper uses the number of confirmed cases per 100,000 people in each country to measure that effectiveness.

#### Independent Variable

The independent variable of this paper is the strategic partnership, which is measured by the closeness of the bilateral partnership. The value is 0 for unestablished partnerships, one for partnerships, two for comprehensive partnerships, three for strategic partnerships, and four for comprehensive strategic partnerships. The higher the value, the stronger the partnership.

#### Moderator Variables

There are three moderator variables in this paper. The first is institutional distance, which reflects the institutional environment differences between China and emerging economies. The measurement method refers to the formula of Kogut and Singh (48), that is, $ID=\sum \left[{\left(I_{kCH}-I_{kj}\right)}^{2}/V_{k}\right]/6$. Among these, *I*_*kCH*_ represents the index value of the k-th institutional dimension of China, *I*_*kj*_ represents the index value of the k-th institutional dimension of emerging economies j, and *V*_*k*_ represents the variance of the index value of the k-th institutional dimension. The data of each institutional dimension comes from six aspects of the Worldwide Governance Indicators (WGI) provided by the World Bank (i.e., voice and accountability, political stability and absence of violence or terrorism, government effectiveness, regulatory quality, rule of law, and control of corruption). The second is cultural distance, which reflects the degree of cultural difference between China and emerging economies. The cultural distance between China and various emerging economies is measured according to the cultural distance measurement index proposed by Kogut and Singh (48). That is, $CD=\sum \left[{\left(J_{kCH}-J_{kj}\right)}^{2}/V_{k}\right]/6$. Among them, *J*_*kCH*_ represents the index value of the k-th cultural dimension of China, *J*_*kj*_ represents the index value of the k-th cultural dimension of emerging economies j, and *V*_*k*_ represents the variance of the index value of the k-th cultural dimension. The data for each cultural dimension comes from https://geert-hofstede.com, which is based on Hofstede's proposed cultural dimensions (i.e., power distance, uncertainty avoidance, individualism vs. collectivism, masculinity vs. femininity, long-term vs. short-term, indulgence vs. restraint). One can obtain a score for each cultural dimension of the economy by entering a country's name. The third is geographical distance, which reflects the geographical proximity between China and emerging economies, measured by the straight-line distance between Beijing, the capital of China, and the capitals of emerging economies.

#### Control Variables

In this paper, five variables are included as control variables from the three aspects of economy, population, and medical care. The first is the closeness of bilateral economic ties, which is measured by the total import and export trade between China and emerging economies; the second is the level of national economic development, which is measured by the GDP per capita of emerging economies; the third is the age structure of the country's population, which is measured by the proportion of the population aged 65 and over to the total population; the fourth is the health status of the national population, which is measured by the incidence of tuberculosis per 100,000 people; the fifth is the national medical and health level, which is measured by the coverage of health services in the infectious diseases index.

The variable definitions and measurement methods in this paper are shown in Table 1.

**Table 1:** Variable definition and assignment.

**Variable type**	**Variable code**	**Variable assignment**	**Data source**
Dependent variable	lnincidence	Confirmed cases per 100,000 persons	Johns Hopkins University New Coronavirus Research Center: https://coronavirus.jhu.edu/
Independent variable	lnrelationship	From 0 to 4, the higher the level of partnership relationship, the higher the value.	Ministry of Foreign Affairs of the People's Republic of China:
Moderator variables	Inid	Institutional distance	The Worldwide Governance Indicators http://info.worldbank.org/governance/wgi/home
	lncd	Cultural distance	https://geert-hofstede.com
	lngd	Geographical distance	CEPII-Database
Control variables	lntrade	Total value of import and export	UN Comtrade Database
	lngdp_per	GDP per capita	World Bank Database
	lnelderly	Population ages 65 and above (% of total population)	World Bank Database
	lntuberculosis	Incidence of tuberculosis (per 100,000 people)	World Bank Database
	lnsci_infectious	UHC Service Coverage sub-index on infectious diseases	WHO website https://www.who.int/data/gho/data/indicators/indicators-index

### Descriptive Statistics

Descriptive statistics and correlations of the variables are shown in Table 2. The statistics show that the variables used in this study are correlated and that there is indeed a relationship between the variables. Moreover, the correlation coefficient between key variables is low, which preliminarily shows that there is no serious problem in multicollinearity between variables. In addition, in the process of regression analysis, we have conducted a variance inflation factor test. The results show that the overall VIF mean and the VIF coefficient of each independent variable do not exceed 10, which further indicates that there is no multicollinearity problem in this paper.

**Table 2:** Descriptive statistics and correlation coefficient matrix of variables.

**Variable**	**Mean**	**Std.** **Dev**.	**(1)**	**(2)**	**(3)**	**(4)**	**(5)**	**(6)**	**(7)**	**(8)**	**(9)**	**(10)**
(1) lnincidence	5.521	2.293	1									
(2) lnrelationship	0.606	0.743	0.0410	1								
(3) lntrade	13.44	3.986	0.146*	0.440***	1							
(4) lngdp_per	8.195	1.131	0.464***	0.135	0.0650	1						
(5) lnelderly	1.903	0.543	0.444***	0.0910	0.0280	0.582***	1					
(6) lntuberculosis	4.087	1.426	−0.371***	0.0560	0.253***	−0.588***	−0.442***	1				
(7) lnsci_infectious	4.145	0.282	0.434***	0.183**	−0.0530	0.716***	0.619***	−0.557***	1			
(8) lngd	8.996	0.561	0.152*	−0.312***	−0.172**	−0.0930	−0.0720	−0.184**	−0.0370	1		
(9) lncd	1.484	0.488	0.0500	−0.373***	−0.526***	−0.361***	−0.216*	−0.124	−0.217*	0.493***	1	
(10) lnid	1.98	0.564	−0.205**	−0.220***	−0.328***	−0.120	−0.0590	0.0740	−0.265***	0.0910	0.0470	1

Standard errors in parentheses: ***p < 0.01, **p < 0.05, *p < 0.1.

## Model Selection and Empirical Result

### Model Selection

In order to test the impact of strategic partnership on the effect of pandemic prevention and control (H1), we employ the following linear estimation model.


(1) $$lnincidence=\beta_{0}+\beta_{1}\;\cdot \;lnrelationship+\delta \;\cdot \;CV+\varepsilon$$

Furthermore, in order to study whether different institutional environments, cultural backgrounds and geographic distances between the two countries have different effects on epidemic prevention and control, we expand model (2), (3) and (4) on the basis of model (1), and explore the moderating effect of institutional ,cultural and geographic distances, respectively:


(2) $$\begin{array}{l} lnincidence=\beta_{0}+\beta_{1}\;\cdot \;lnrelationship+\beta_{2}\;\cdot \;lnid\\ \;+\beta_{3}\;\cdot \;\left(lnrelationship\cdot lnid\right)+\delta \;\cdot \;CV+\varepsilon \end{array}$$

(3) $$\begin{array}{l} lnincidence=\beta_{0}+\beta_{1}\;\cdot \;lnrelationship+\beta_{2}\;\cdot \;lncd\\ \;+\beta_{3}\;\cdot \;\left(lnrelationship\cdot lncd\right)+\;\cdot \cdot \;CV+\varepsilon \end{array}$$

(4) $$\begin{array}{l} lnincidence=\beta_{0}+\beta_{1}\;\cdot \;lnrelationship+\beta_{2}\;\cdot \;lngd\\ \;+\beta_{3}\;\cdot \;\left(lnrelationship\cdot lngd\right)+\delta \;\cdot \;CV+\varepsilon \end{array}$$

Among these, the independent variable lnincidence (i.e., the incidence rate) represents the effect of pandemic prevention and control. The independent variable lnrelationship (i.e., the strategic partner relationship) represents the closeness of the bilateral partnership. lnid, lncd, and lngd are three moderator variables, which are geographic distance, cultural distance, and institutional distance. CV represents the set of control variables that affect the impact of pandemic prevention and control, including lntrade, lngdp_per, lnelderly, lntuberculosis, and lnsci_infectious, and ε represents the random disturbance term.

### Empirical Result

Hypothesis 1 of this paper is that the establishment of strategic partnership is conducive to improving the effect of pandemic prevention and control, and the incidence of COVID-19 is used as a reverse indicator to measure that effect. Table 3 reports the baseline regression results. Model (1) is a basic model with only control variables, while model (2) adds the independent variable based on model (1). The results show that partnership has a significant negative correlation with the incidence of COVID-19 (β = −0.5996, *p* < 0.05), which suggests that the closer the partnership between China and emerging economies, the lower the incidence of COVID-19 in emerging economies. Therefore, the establishment of strategic partnerships will help emerging economies to achieve better pandemic prevention and control effects. Hypothesis 1 is supported.

**Table 3:** Baseline regression results.

	**(1)**	**(2)**
**Variables**	**lnincidence**	**lnincidence**
lnrelationship		−0.5996**
		(0.2320)
lntrade	0.1506***	0.2146***
	(0.0546)	(0.0588)
lngdp_per	0.5358**	0.5283**
	(0.2180)	(0.2153)
lnelderly	0.6774*	0.6581*
	(0.3753)	(0.3697)
lntuberculosis	−0.1253	−0.1010
	(0.1543)	(0.1543)
lnsci_infectious	0.6320	1.0137
	(0.8624)	(0.8639)
Constant	−4.3027	−6.3952*
	(3.2232)	(3.3134)
Observations	140	139
R-squared	0.3311	0.3640

Standard errors in parentheses: ***p < 0.01, **p < 0.05, *p < 0.1.

### Robustness Test

In order to test the robustness of the baseline regression results, we use the method of replacing key variables. We use the case fatality (lncase_fatality) to replace the original incidence (lnincidence), thereby measuring the effectiveness of pandemic prevention and control, and use the number of visits (lnvisit) between state leaders as the proxy variable of the partnership (lnrelationship), while other variables remain unchanged. It can be seen from Table 4 that the coefficient of independent variables is significantly negative (*p* < 0.05), indicating that the estimated result is still robust. In addition, in the subsequent moderating effects test (Table 5), after the moderator variables and cross terms are included in the regression model, except for the significant level in model (6), the coefficient symbols and significant levels of independent variables in other models have not changed significantly. This shows that the estimation result is robust.

**Table 4:** Robustness test.

	**(3)**	**(4)**
**Variables**	**lncase_fatality**	**lncase_fatality**
lnvisit		−0.1047**
		(0.0525)
lntrade	0.0611***	0.0767***
	(0.0136)	(0.0156)
lngdp_per	−0.1794***	−0.1807***
	(0.0543)	(0.0537)
lnelderly	0.2841***	0.3198***
	(0.0935)	(0.0942)
lntuberculosis	−0.0714*	−0.0612
	(0.0384)	(0.0384)
lnsci_infectious	−0.3085	−0.2956
	(0.2148)	(0.2125)
Constant	2.5634***	2.3332***
	(0.8028)	(0.8024)
Observations	140	140
R-squared	0.2061	0.2292

Standard errors in parentheses: ***p < 0.01, **p < 0.05, *p < 0.1.

**Table 5:** Moderating effects test.

	**(5)**	**(6)**	**(7)**
**Variables**	**lnincidence**	**lnincidence**	**lnincidence**
lnrelationship	−0.5039**	−0.2874	−0.5942**
	(0.2346)	(0.3076)	(0.2609)
lnid	−0.4244		
	(0.3276)		
lnrelationship * lnid	0.7851*		
	(0.3998)		
lncd		−0.5483	
		(0.6035)	
lnrelationship * lncd		1.4345**	
		(0.6693)	
lngd			0.3948
			(0.4019)
lnrelationship * lngd			0.8029*
			(0.4697)
lntrade	0.1970***	−0.0222	0.3251***
	(0.0601)	(0.1328)	(0.0904)
lngdp_per	0.5146**	0.2064	0.5065**
	(0.2143)	(0.3108)	(0.2207)
lnelderly	0.7858**	1.1085***	0.4431
	(0.3729)	(0.4110)	(0.3887)
lntuberculosis	−0.0838	−0.4846**	−0.0477
	(0.1529)	(0.2030)	(0.1619)
lnsci_infectious	0.8495	−0.8007	1.3079
	(0.9043)	(1.3266)	(0.8720)
Constant	−6.0153*	8.3801	−9.1554***
	(3.5049)	(5.2242)	(3.4948)
Observations	139	64	128
R-squared	0.3871	0.4060	0.3902

Standard errors in parentheses: ***p < 0.01, **p < 0.05, *p < 0.1.

### Moderating Effects Test

Hypotheses 2, 3, and 4 in this paper respectively assume that the positive effect of establishing strategic partnerships on COVID-19 prevention and control may be influenced by differences of institutional, cultural, and geographical distance. In this paper, we constructed the interaction terms of one independent variable (lnrelationship) and three moderator variables (lnid, lncd, and lngd). Moreover, in this paper, we have constructed the independent variable lnrelationship and the interaction terms of the three moderator variables. Model (5), (6) and (7), respectively incorporate moderator variables and interaction terms based on model (2), to investigate the effect of differences in multiple distances on the impact of establishing strategic partnerships for pandemic prevention and control. From the regression results in Table 5, we can see that the coefficient of lnrelationship^*^lnid is significantly positive [β = 0.7851, *p* < 0.1, model (5)], the coefficient of lnrelationship^*^lncd is significantly positive [β = 1.4345, *p* < 0.05, model (6)], and the coefficient of lnrelationship^*^lngd is also significantly positive [β = 0.8029, *p* < 0.1, model (7)]. The signs of the three interaction terms are all opposite to the independent variable, indicating that institutional, cultural, and geographical distance have negative moderating effects on the relationship between the establishment of strategic partnerships and pandemic prevention and control in emerging economies. When the institutional, cultural, and geographical distances between countries are relatively far, the positive impacts of establishing strategic partnerships on prevention and control in emerging economies will be weakened. Thus, hypotheses 2, 3, and 4 of this paper are supported.

## Further Discussion

In this paper, we find that the establishment of strategic partners is positively correlated with the effectiveness of pandemic prevention and control. There are three main reasons for this. First, close partnerships between countries reduce the likelihood of public health securitization. Securitization occurs when a threatening issue is sufficiently prominent to allow authorities to take approaches that they deem most appropriate to deal with the issue (49). The strategic partnership will prompt a country to consider bilateral relations from multiple perspectives, thus avoiding the use of non-transparent competition in response to the pandemic crisis, which would affect cooperation. Scientists can conduct in-depth cooperation in, for example, virus detection and vaccine development without being restricted by political situations and policies based on national interests (19). Second, strategic partnerships will encourage the two sides to elevate cooperation in prevention and control to the height of political diplomacy, attaching importance to developing scientific globalism rather than scientific nationalism, and avoiding unnecessary duplication of research work and competition between research centers. Compared with scientific globalism, whose mission is to promote knowledge progress and scientific openness, scientific nationalism pays more attention to its own interests rather than global interests, and regards scientific and technological progress as a way to ensure national security and demonstrate national prestige (50). The establishment of strategic partnerships between countries will not only help countries with relatively weak medical and health research capabilities to obtain the right to use drugs and vaccines, thereby reducing the number of infections and deaths under COVID-19, but will also prompt countries with relatively strong medical and health research capabilities to carry out in-depth international cooperation and share achievements in drug and vaccine research and development, thereby providing key information support for the implementation of scientific and targeted pandemic control measures for the benefit of the world (51). Third, Compared with developed countries, most of the emerging economies have an unsound market economy. Enterprises in these countries often promote enterprise growth through political connections (52). Therefore, the establishment of strategic partnerships between governments can promote trade between enterprises of both sides, which is not only beneficial to form a more stable community of interests, but also easier to form a consensus in dealing with public crises.

From the perspective of different dimensions in distance, we find that multiple distances—institutional, cultural, geographical—have negative moderating effects on the relationship between the establishment of strategic partnerships and the effect of prevention and control. It is not surprising that these multiple distances have significant moderating effects. On the one hand, despite the many similarities among countries during the pandemic, measures to deal with the spread of COVID-19 and priorities in prevention and control may differ due to differences in countries' geographical locations, cultures, and institutions (24), which will lead to different effects of establishing strategic partnerships on prevention and control. On the other hand, the COVID-19 pandemic has changed public health diplomacy from a mindset centered on development assistance to a shared global goal that can only be achieved by all countries working together (53). Generally speaking, geographical proximity, and cultural and institutional similarity can prompt countries to take the lead in establishing a regional cooperation mechanism for the prevention and control of infectious diseases and to carry out closer cooperation, such as strengthening exchanges on public health and cooperation on information, promoting telemedicine cooperation between hospitals on both sides, carrying out projects in medical and health service cooperation, and advancing cross-border cooperation in joint pandemic prevention and control, so as to effectively control the spread of the pandemic.

## Conclusion

In this paper, we have examined the impact of strategic partnerships among emerging economies on the effectiveness of COVID-19 prevention and control. We have studied the relationship between incidence and strategic partnership among 165 emerging economies. The results indicate that a good relationship in political cooperation is of great importance for countries to work together to fight the pandemic and slow down the spread of the virus. On this basis, we further examine the adjustment mechanisms of institutional, cultural, and geographical distance between emerging economies on this driving effect, and the results show that all three distances will weaken this driving effect.

Based on the above research conclusions, we put forward the following suggestions for the urgent needs of pandemic prevention and control:

(1) Political mutual trust and strategic mutual trust are the cornerstones of cooperation among countries. We should take the joint prevention and control of the pandemic situation as an opportunity to promote coordination and cooperation among countries. By establishing a solid foundation of political mutual trust among countries, such as strategic partnership and high-level leaders' visits for exchange, strengthening cooperation based on the purpose of mutual benefit and win-win, adhering to the principles of openness, tolerance and seeking common ground while reserving differences, and promoting pragmatic cooperation on major issues of difference.
(2) Governments should establish high-level systems and mechanisms, explore effective anti-pandemic cooperation mechanisms and extend them widely. Countries can try to establish bilateral cooperation first, then gradually move toward multilateral cooperation, regional cooperation, and global cooperation, and improve the global system for the governance of public health safety.
(3) Governments should also promote multi-level collaboration among non-governmental organizations in various countries. They should strengthen the cultural exchange mechanism, carry out non-governmental cultural exchange activities, enhance cultural identity, and promote people's communication. In terms of cooperation on scientific research, universities and research institutions are encouraged to conduct academic exchanges and project cooperation, establish a joint training mechanism for medical talent, and accelerate the research and development of vaccines and anti-pandemic drugs. Countries should give full play to the role of enterprises. Whether it is the supply and transportation of materials for prevention and control or the production of vaccines, enterprises need to participate. Enterprises need to build pandemic bases for the production of prevention-related material such as masks, vaccines, and protective clothing in local areas, thereby reducing the shortage of these materials caused by insufficient production capacity and inefficient transportation.

The development of today's world is inseparable from emerging economies. According to IMF statistics, emerging economies have contributed nearly 50% of world economic growth in the past 10 years. A global multi-polarization system is taking shape. It is foreseeable that the official development assistance from developed countries to developing countries will gradually weaken, and emerging economies need to further strengthen open cooperation in the fields of politics, economy, science and technology, get rid of excessive dependence on developed countries, and improve their international voice.

## Data Availability Statement

The original contributions presented in the study are included in the article/supplementary material, further inquiries can be directed to the corresponding author.

## Author Contributions

All authors listed have made a substantial, direct, and intellectual contribution to the work and approved it for publication.

## Conflict of Interest

The authors declare that the research was conducted in the absence of any commercial or financial relationships that could be construed as a potential conflict of interest.

## Publisher's Note

All claims expressed in this article are solely those of the authors and do not necessarily represent those of their affiliated organizations, or those of the publisher, the editors and the reviewers. Any product that may be evaluated in this article, or claim that may be made by its manufacturer, is not guaranteed or endorsed by the publisher.

## Footnotes

^1^Data from the Johns Hopkins University New Coronavirus Research Center: https://coronavirus.jhu.edu/.

^2^Data source: Ministry of Foreign Affairs of the People's Republic of China: www.mfa.gov.cn/web/gjhdq_676201/gj_676203/yz_676205/.
